# Critical Considerations in Calling Disease-Causing *EDAR* Mutations in Nonsyndromic Oligodontia

**DOI:** 10.3390/jcm13237328

**Published:** 2024-12-02

**Authors:** Youn Jung Kim, Se-Young Gu, Wonseon Chae, Seon Hee Kim, Jung-Wook Kim

**Affiliations:** 1 Department of Pediatric Dentistry, School of Dentistry & Dental Research Institute, Seoul National University, Seoul 03080, Republic of Korea; ykim71@snu.ac.kr (Y.J.K.); likealike@naver.com (S.-Y.G.); cws50055752@gmail.com (W.C.); shsunnyten19@gmail.com (S.H.K.); 2 Department of Molecular Genetics, School of Dentistry & Dental Research Institute, Seoul National University, Seoul 03080, Republic of Korea

**Keywords:** oligodontia, *EDAR*, nonsyndromic, recessive, mutation, luciferase assay

## Abstract

**Background/Objectives:** Oligodontia, the absence of six or more teeth excluding third molars, is a rare genetic condition, unlike hypodontia (missing one or more teeth), which is a relatively common human disease. **Methods:** To identify the genetic etiology of nonsyndromic oligodontia (NSO) families, we performed mutational analysis and investigated the functional effects of identified *EDAR* mutations. Whole-exome sequencing was conducted on recruited families with NSO. Bioinformatic analysis identified mutations in oligodontia-causing candidate genes, which were confirmed by Sanger sequencing and segregation within families. The impact of *EDAR* mutations on the EDA signaling pathway was assessed using luciferase activity analysis. **Results:** *EDAR* mutations were identified in three NSO families. A homozygous missense *EDAR* mutation (NM_022336.4: c.319A>G p.(Met107Val)) was found in the singleton proband of family 1. The proband of family 2 carried compound heterozygous *EDAR* mutations: a maternal missense mutation (c.319A>G p.(Met107Val)) and a paternal missense variant (c.1270G>A p.(Val424Met)). The proband of family 3 had heterozygous *EDAR* mutations: a maternal missense mutation (c.389T>A p.(Ile130Asn)) and paternal intronic variants in cis (c.[357-4G>A;440+50C>T]). Luciferase assays confirmed reduced transcriptional activity for all identified missense mutations, while splicing assays revealed altered splicing patterns. **Conclusions:** In conclusion, recessive *EDAR* mutations, including novel ones, were identified in NSO families, and their pathological mechanism was explored through transcriptional activity measurements.

## 1. Introduction

Like most mammals, humans have two sets of dentition—the deciduous and permanent sets. The primary dentition, which consists of 20 milk teeth, usually begins to erupt at 6 months and is completed by 24 months [1]. Excluding the third molars, the permanent dentition comprises 28 teeth and begins to erupt around the age of 6, replacing the deciduous teeth.

The absence of one or more teeth is termed hypodontia and is a relatively common genetic disorder [2]. However, oligodontia, defined as the absence of six or more teeth except for third molars, is rare and may require comprehensive long-term treatment [3,4]. Oligodontia can occur as an isolated form without any other symptoms. However, given the ectodermal origin of tooth development, missing teeth could also be a phenotype of a syndrome, such as ectodermal dysplasia [5].

A search using the term “oligodontia” in the OMIM (Online Mendelian Inheritance in Man) [6], an online catalog of human genes and genetic disorders, yields 110 results as of this writing. The results include overlapping genes and disease categories due to overlapping phenotypes and allelic disorders. There are 11 selective tooth agenesis categories in this database (Table 1).

The EDA/EDAR/NFκB pathway is one of the major signaling pathways involved in tooth development [7]. EDA is a type II transmembrane protein with a large C-terminal extracellular domain with TNF homology, but furin cleavage produces a secreted form [8]. The cleaved EDA fragments form a homotrimer, which interacts with the EDA receptor (EDAR). Binding to the EDAR adapter (EDARADD) forms an EDA/EDAR/EDARADD complex and activates downstream NFκB signaling, which is an important regulator of many developmental processes, including tooth development [9].

In this study, we recruited families with nonsyndromic oligodontia (NSO) and performed mutational analysis using candidate gene sequencing and whole-exome sequencing. *EDAR* mutations were identified, with the effects of the mutations being investigated.

## 2. Materials and Methods

### 2.1. Study Subjects

Families with NSO were recruited with informed consent. The nature of the study was explained to all participating individuals before obtaining consent. Peripheral blood or saliva samples were collected for DNA isolation. The study protocol was independently reviewed and approved by the Institution Review Board at the Seoul National University Dental Hospital.

### 2.2. Isolation of Genomic DNA and Whole-Exome Sequencing

Genomic DNA was isolated from 2 mL of peripheral blood or saliva samples using the NucleoSpin Blood L Kit (Macherey-Nagel GmbH & Co., Düren, Germany). Candidate gene sequencing of the *MSX1* gene was performed for families 1 and 3, as previously described [10]. DNA from the proband of each family was submitted for whole-exome sequencing (BGI, Shenzhen, China). After exome capturing with Agilent’s SureSelect Target Enrichment System (Agilent Technologies, Santa Clara, CA, USA), 100 bp paired sequencing reads were generated.

### 2.3. Bioinformatic Analysis

Raw data were filtered to remove low-quality sequences using SOAPnuke software (Release 2.1.6, BGI). Filtered sequencing reads were trimmed to remove adapter sequences using Trimmomatic v0.33 [11]. The trimmed reads were aligned to the hg38 reference human genome assembly using bwa 0.6.2 [12]. A list of sequence variants, including single nucleotide changes and small insertions/deletions, was obtained after applying a series of bioinformatic programs (samtools v1.21, gatk v4.6.1.0, bcftools v1.21, and annovar) [13,14,15]. The dbSNP build 150 was used to annotate the sequence variants, which were then filtered with a minor allele frequency of 0.1. Mutational effects were predicted in silico using PolyPhen-2 (http://genetics.bwh.harvard.edu/pph2/, accessed on 20 June 2024) [16], Mutation Taster (https://www.mutationtaster.org/, accessed on 20 June 2024) [17], and the CADD score (version 1.7, https://cadd.gs.washington.edu/, accessed on 20 June 2024) [18].

### 2.4. Confirmation of the Identified Variants

Identified variants and segregation within the family were confirmed by Sanger sequencing (Macrogen, Seoul, Republic of Korea). The identified novel *EDAR* mutation was submitted to the ClinVar database (https://www.ncbi.nlm.nih.gov/clinvar/, accessed on 27 July 2024, Accession ID: SCV005088567).

### 2.5. In Vitro Minigene Splicing Assay

A genomic fragment (367 bp) containing exon 5 of the *EDA* gene was amplified using DNA from the proband of family 3 (sense primer: 5′-CTGAAACCCCAAGGAAACTG-3′ and antisense primer: 5′-ACTGTGATTCACCTTGTCCAG-3′) with the Pfu plus master mix (Elpis-Biotech, Daejeon, Republic of Korea) and cloned into the TOPcloner blunt V2 vector (Enzynomics, Daejeon, Republic of Korea). The sequence of each allele was confirmed, and the fragments were subcloned into the pSPL3 splicing vector after EcoRI restriction enzyme cutting. Clones with the correct sequence and direction were selected by directional colony PCR, and the sequences were confirmed by direct sequencing. The maternal allele had a missense variant in exon 5 (NM_022336: c.389T>A p.(Ile130Asn)), and the paternal allele had two intronic variants (c.[357-4G>A;440+50C>T]). To test the effect of each variant on pre-mRNA splicing, PCR mutagenesis was performed to restore variants to the wildtype sequence with the following primers (sense primer: 5′-CCGAGGAACATCTATGGCATG-3′ and antisense primer: 5′-CATGCCATAGATGTTCCTCGG-3′ for c.389T>A; sense primer: 5′-TCTCTCCCCCGTAGCTACTAC-3′ and antisense primer: 5′-GTAGTAGCTACGGGGGAGAGA-3′ for c.357-4G>A; sense primer: 5′-AGCACGGATCCCTTTCACTAC-3′ and antisense primer: 5′-GTAGTGAAAGGGATCCGTGCT-3′ for c.440+50C>T). The sequences of each vector were confirmed by direct sequencing. COS7 cells were transiently transfected with the pSPL3 vectors, total RNA was isolated after 36 h, and cDNA was synthesized. Amplification bands from RT-PCR (SD6 sense primer: 5′-TCTGAGTCACCTGGACAACC-3′ and SA2 antisense primer: 5′-ATCTCAGTGGTATTTGTGAGC-3′) were excised from a polyacrylamide electrophoresis (PAGE) gel and characterized by cloning and sequencing.

### 2.6. Cloning of EDAR Expression Vector

Myc-tagged human *EDAR* cDNA (1395 bp) with 5′ HindIII and 3′ XbaI restriction sites was synthesized (Macrogen, Seoul, Republic of Korea) and initially cloned into the pMG-Amp vector. The cDNA fragment was then excised by HindIII and XbaI double digestion and subcloned into the pcDNA3.1(+) mammalian expression vector. Desired mutations were introduced into this expression vector using PCR-based site-directed mutagenesis (Table 2). For co-transfection experiments, an EDA pcDNA3.1(+) mammalian expression vector was used.

### 2.7. Luciferase Assay

A firefly luciferase reporter plasmid containing NFκB enhancer elements was used to quantify the transcriptional activity of EDAR. HEK293 cells were co-transfected with this reporter plasmid and a constitutive Renilla luciferase plasmid. Cells in 12-well plates were transfected with a total of 1 μg of plasmid mixture (0.3 μg of EDA expression pcDNA3.1(+), 0.3 μg of EDAR expression pcDNA3.1(+), 0.2 μg of NFκB reporter plasmid, and 0.2 μg of pRL-TK plasmid). Transfection was performed using GenJet (SignaGen, Frederick, MD, USA) following the manufacturer’s protocol. At 72 h post-transfection, the culture medium was removed, and cells were washed with PBS. Cells were then lysed on ice using 1× Passive Lysis buffer (Promega, Madison, WI, USA). After 30 min of incubation on ice, cell lysates were collected by centrifugation at ~13,000× *g* for 15 min at 4 °C. Renilla and firefly luciferase activities were sequentially measured using a Dual Luciferase Assay Kit (Promega) according to the manufacturer’s instructions. Renilla luciferase activity was used as an internal control. Luciferase activity was determined using a SynergyH1 microplate reader (BioTek, Winooski, VT, USA). The experiments were conducted in triplicate, and the results were subjected to statistical analysis using one-way ANOVA, followed by Tukey’s honestly significant difference test.

### 2.8. Protein Structure Analysis

The 3D structural modeling of the EDAR fragment interacting with EDA was performed using PyMOL software (PyMOL Molecular Graphics System, Version 3.0.5, Schrödinger, LLC., DeLano Scientific, Palo Alto, CA, USA; http://www.pymol.org/, accessed on 20 September 2024) [19]. Protein Data Bank (PDB: https://www.rcsb.org/, accessed on 20 September 2024) coordinates (1RJ7 and 7X9G) were used for the modeling [20,21].

## 3. Results

### 3.1. Family 1

**Figure 1:**
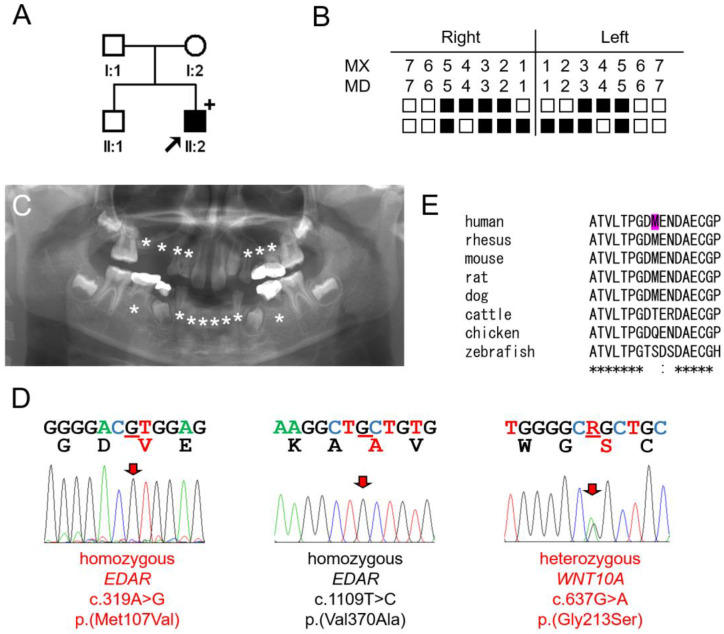
Pedigree, panoramic radiograph, homolog alignment, and sequencing chromatograms of family 1. (**A**) Pedigree of family 1. Black symbol indicates affected individual, and the proband is indicated by a black arrow. Mother and father could not participate in the study. The plus sign above the symbol indicates the participating individual. (**B**) Summary chart of missing teeth of the proband. He is missing 15 permanent teeth (black filled symbol means missing tooth). (**C**) Panoramic radiograph of the proband at age 9 years and 8 months. Missing permanent teeth are indicated with asterisks. (**D**) Sequencing chromatograms of the proband. Left one shows a homozygous *EDAR* c.319A>G p.(Met107Val) mutation. Middle one shows a homozygous *EDAR* c.1109T>C p.(Val370Ala) variant. Right one shows a heterozygous *WNT10A* c.637G>A p.(Gly213Ser) mutation. Mutated nucleotides are indicated with red arrows and underlined with red bars (R = A and G). Amino acids encoded are shown under the nucleotide sequences. Mutated amino acids are shown in red color. Amino acids shown in black color represents wildtype amino acid sequences. (**E**) Homolog alignment. The methionine at the 107 codon position is partially conserved among vertebrates and indicated with magenta color. The asterisk under the alignment indicates the conserved amino acid position (human: NP_071731.1, rhesus: XP_014968589.2, dog: XP_038406764.1, cattle: XP_005212787.1, mouse: NP_034230.1, rat: NP_001178828.1, chicken: NP_001012629.1, and zebrafish: NP_001108536.2).

The proband of family 1 was a nine-year and eight-month-old boy, the second child in his family, missing fifteen permanent teeth (Figure 1). His second permanent molars were developing in all four quadrants, but the development of third molars could not yet be determined. His deciduous dentition was reportedly complete, without any missing teeth. There was no remarkable past medical history, including during pregnancy and delivery. In addition to the missing teeth, the maxillary left lateral incisor was a peg lateralis. Other family members had no missing teeth. There were no other health problems, including symptoms related to ectodermal dysplasia.

Whole-exome sequencing revealed three interesting variants in genes related to oligodontia. The proband had two homozygous *EDAR* variants (NM_022336.4:c.319A>G p.(Met107Val) and c.1109T>C p.(Val370Ala)), and a heterozygous *WNT10A* variant (NM_025216.3:c.637G>A p.(Gly213Ser)). All three variants were previously reported, but the mutational results and in silico predictions of *EDAR* variants were inconsistent.

The p.(Met107Val) variant was predicted as benign in all three predictions (medium value in CADD 1.7), and the minor allele frequency (MAF) was relatively high (Table 3). Also, conservation among species was not complete. However, this variant has been reported as a disease-causing mutation in three autosomal dominant oligodontia families [22]. The proband of family 1 had this variant in a homozygous condition.

The p.(Val370Ala) variant showed variable in silico predictions: Mutation Taster predicted it as a polymorphism, while PolyPhen-2 and CADD 1.7 presented more pathogenic predictions. The minor allele frequency (MAF) was high at 0.03989 globally; however, it reached 0.8451 in East Asian and 0.4294 in Admixed American genetic ancestry groups (https://gnomad.broadinstitute.org/variant/2-108897145-A-G?dataset=gnomad_r4, accessed on 27 July 2024). Consequently, it is not a minor allele in these populations. Strong genetic selection for this variant in East Asia has been reported, with its effects characterized in vitro and through transgenic and knock-in mouse models [23,24]. The MAF was 0.47 in Latino cohorts, with recent studies suggesting associations with metabolic syndrome and breast density [25]. This variant has also been linked to crown shape and root morphology [26,27]. In mice, the p.(Val370Ala) variant resulted in higher signal output and increased hair thickness, potentially contributing to the thicker and straighter hair typical of East Asian populations [28]. Although the proband of family 1 was homozygous for this variant, it seemed unlikely to contribute to the disease condition.

The proband also had a heterozygous variant (NM_025216.3:c.637G>A p.(Gly213Ser)) in exon 3 of the *WNT10A* gene [29]. This mutation was previously reported in a Korean oligodontia family with autosomal recessive inheritance [30]. In that family, the proband had compound heterozygous *WNT10A* mutations (paternal c.364A>T p.(Ile122Phe) and maternal c.637G>A p.(Gly213Ser)). However, both parents who had a heterozygous mutation did not present any symptoms.

Therefore, it appears that the homozygous p.(Met107Val) *EDAR* mutation is the primary disease-causing mutation, and the heterozygous p.(Gly213Ser) *WNT10A* mutation is a contributing factor that may worsen the degree of symptoms [31,32].

### 3.2. Family 2

**Figure 2:**
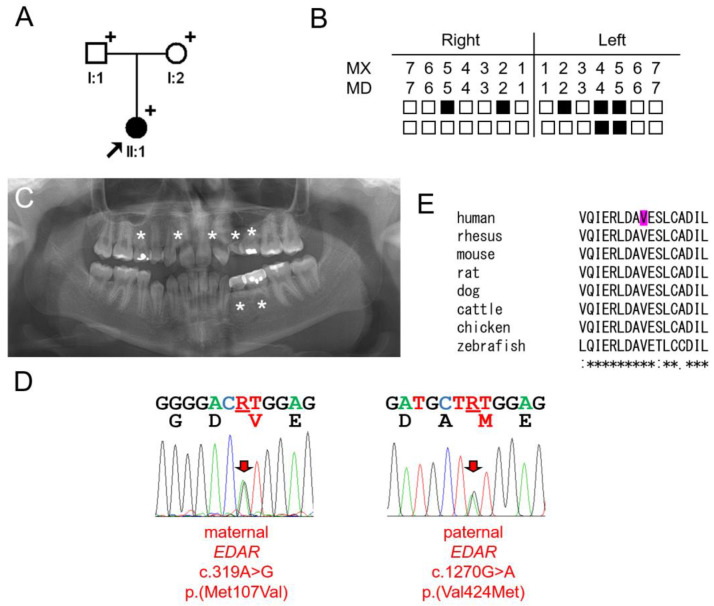
Pedigree, panoramic radiograph, homolog alignment, and sequencing chromatograms of family 2. (**A**) Pedigree of family 2. Black symbol indicates affected individual, and the proband is indicated by a black arrow. Plus signs above the symbols indicate participating individuals. (**B**) Summary chart of missing teeth of the proband. She is missing 7 permanent teeth (black filled symbol means missing tooth). (**C**) Panoramic radiograph of the proband at age 13 years and 11 months. Missing permanent teeth are indicated with asterisks. (**D**) Sequencing chromatograms of the proband. Left one shows a maternal *EDAR* c.319A>G p.(Met107Val) mutation. Right one shows a paternal *EDAR* c.1270G>A p.(Val424Met) mutation. Mutated nucleotides are indicated with red arrows and underlined with red bars (R = A and G). Amino acids encoded are shown under the nucleotide sequences. Mutated amino acids are shown in red color. Amino acids shown in black color represents wildtype amino acid sequences. (**E**) Homolog alignment. The valine at the 424 codon position is completely conserved among vertebrates and indicated with magenta color. The asterisk under the alignment indicates the conserved amino acid position (protein reference sequences are the same as in the Figure 1 legend).

The proband of family 2 was a six-year and five-month-old girl from a non-consanguineous family (Figure 2). She was missing deciduous maxillary lateral incisors and seven permanent teeth. The third molars had not yet developed. Apart from the oligodontia, there was no other remarkable past medical history, and no symptoms related to ectodermal dysplasia. Her mother had no missing teeth. However, both maxillary lateral incisors of the father were peg lateralis.

Mutational screening revealed compound heterozygous *EDAR* mutations: a maternal missense mutation (c.319A>G p.(Met107Val)) and a paternal missense variant (c.1270G>A p.(Val424Met)). The paternal mutation was predicted as pathogenic by all three programs used for analysis. It had not been previously reported and was not listed in the gnomAD and dbSNP databases. The valine at codon position 424 was highly conserved among vertebrates.

### 3.3. Family 3

**Figure 3:**
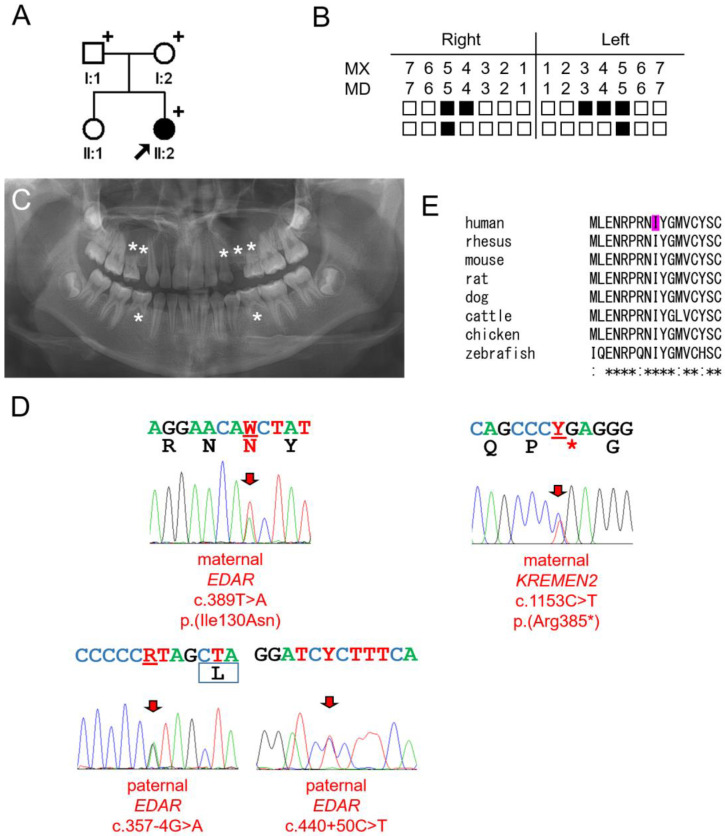
Pedigree, panoramic radiograph, homolog alignment, and sequencing chromatograms of family 3. (**A**) Pedigree of family 3. Black symbol indicates affected individual, and the proband is indicated by a black arrow. Plus signs above the symbols indicate participating individuals. (**B**) Summary chart of missing teeth of the proband. She is missing 7 permanent teeth (black filled symbol means missing tooth). (**C**) Panoramic radiograph of the proband at age 13 years 3 months. Missing permanent teeth are indicated with asterisks. The third molars are developing in all quadrants. (**D**) Sequencing chromatograms of the proband. Left one shows a maternal *EDAR* c.389T>A p.(Ile130Asn) mutation. Lower ones show paternal *EDAR* c.[357-4G>A;440+50C>T] variants. Right one shows a maternal *KREMEN2* c.1270C>T p.(Arg424*) mutation. Mutated nucleotides are indicated with red arrows and underlined with red bars. Amino acids encoded are shown under the nucleotide sequences (W = A and T, R = A and G, and Y = C and T). Mutated amino acids are shown in red color. Amino acids shown in black color represents wildtype amino acid sequences. The box under the nucleotide sequences indicates exon 5. (**E**) Homolog alignment. The isoleucine at the 130 codon position is completely conserved among vertebrates and indicated with magenta color. The asterisk under the alignment indicates the conserved amino acid position (protein reference sequences are the same as in the Figure 1 legend).

The proband of family 3 was a nine-year and five-month-old girl from a non-consanguineous family (Figure 3). She was the second child and was missing seven permanent teeth. She had no remarkable past medical history, and there were no symptoms related to ectodermal dysplasia in her hair, skin, or nails. A panoramic radiograph at age 13 years and 3 months showed developing third molars in all quadrants. The father and sister did not have any missing teeth; however, the mother was missing the right mandibular second premolar, and her maxillary lateral incisors were peg lateralis bilaterally.

Whole-exome sequencing revealed several heterozygous *EDAR* variants and a heterozygous *KREMEN2* variant. The proband carried a maternal missense *EDAR* mutation (c.389T>A p.(Ile130Asn)) and paternal intronic *EDAR* variants in cis (c.[357-4G>A;440+50C>T]). The isoleucine at codon position 130 was highly conserved among vertebrates and was predicted as pathogenic by all three programs used for analysis. This mutation is listed in dbSNP as rs764524798; however, the allele frequency is very low (two heterozygotes among 1,461,888 alleles in the gnomAD database).

The proband also had a heterozygous variant in the *KREMEN2* gene (NM_172229.3: c.1270C>T p.Arg424*). *KREMEN2* encodes a high-affinity dickkopf homolog 1 (DKK1) transmembrane receptor involved in wingless (WNT)/beta-catenin signaling, another major pathway in tooth development [33,34]. This opal nonsense mutation is in the last exon and would likely escape nonsense-mediated mRNA decay, producing a truncated protein of 423 amino acids instead of the normal 462 amino acids. The mutation was predicted as pathogenic with a CADD 1.7 score of 38. This variant is listed in dbSNP as rs1245333253 but is reported in only 1 allele among 161,244 alleles.

**Figure 4:**
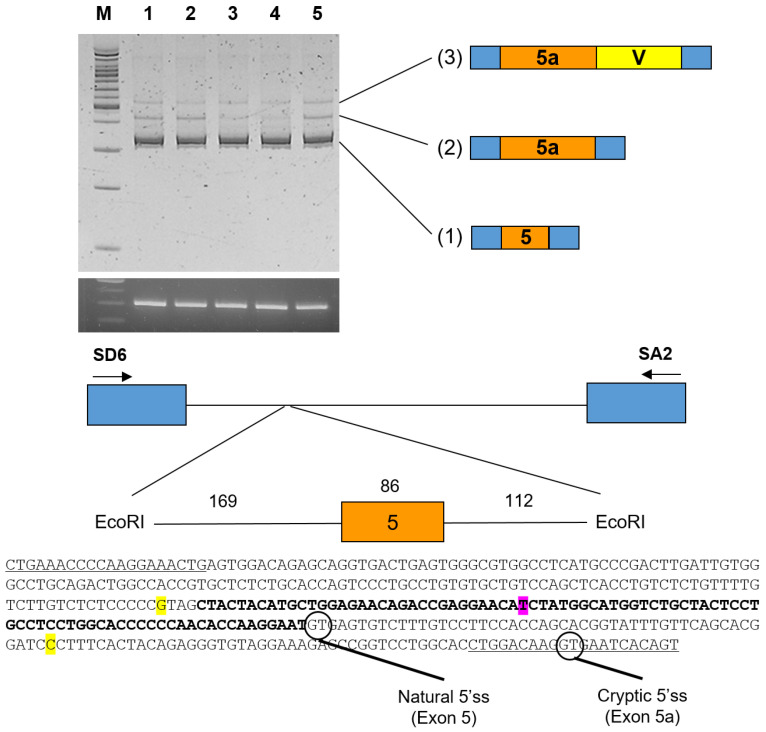
In vitro splicing. A genomic fragment including exon 5 of the *EDAR* gene was subcloned into the pSPL3 vector using EcoRI restriction endonuclease digestion. Boxes indicate exons, and horizontal lines indicate introns. The number of the exon is in the box, and the length of the exon is shown above the box. The length of the intron is shown above the horizontal line. Locations of the primer binding sites are indicated with arrows (sense and antisense). PAGE gel image of the splicing assay is shown on the top. Agarose gel image of *GAPDH* PCR is shown below. Left lane is the DNA ladder (M). Lane 1 is wildtype without any variant. Lane 2 is the maternal allele with a c.389T>A mutation. Lane 3 is the paternal allele with c.[357-4G>A;440+50C>T]. Lane 4 is with only c.440+50C>T and lane 5 is with only c.357-4G>A. Characterization of each band revealed: (1) a transcript with exon 5 only, (2) a transcript with exon 5 and 100 bp intron 5 using cryptic 5′ splicing site (ss), and (3) a transcript with the pSPL3 vector sequence (V) using the splicing site in the pSPL3 vector. In lanes 3 and 4, expression of transcript (2) was very weak. Intronic variants are shown in yellow, while exonic variant is shown in magenta.

The effect of the paternal intronic variants was tested using an in vitro splicing assay with a minigene including only exon 5. Due to the long lengths of intron 4 (5744 bp) and intron 5 (10,603 bp), only a small fragment including exon 5 was cloned and analyzed (Figure 4). Characterization of each transcript revealed a minor cryptic 5′ splicing site in intron 5, which added 100 bp of intron 5 sequences to the exon 5 sequence. This band was present in the wildtype and maternal alleles. Expression of this transcript was greatly reduced in the paternal allele. Mutagenesis testing showed that this reduction was mostly due to the c.440+50C>T variant.

### 3.4. Luciferase Assay

**Figure 5:**
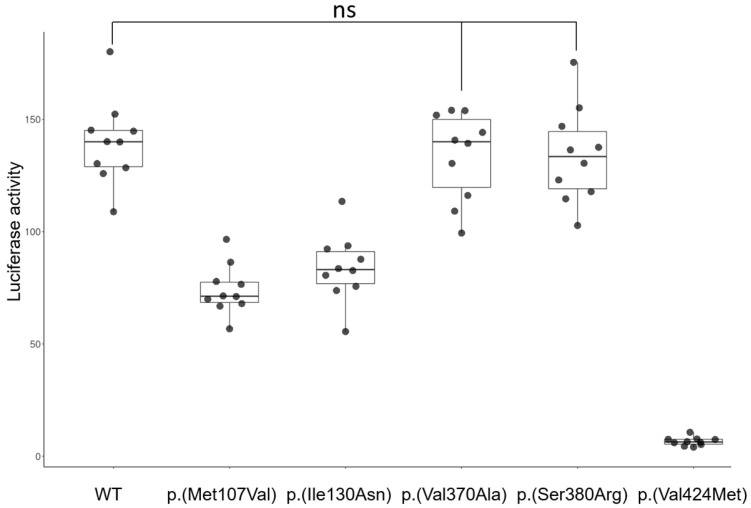
Luciferase assay. Relative luciferase activity of wildtype (WT), p.(Met107Val), p.(Ile130Asn), p.(Val370Ala), p.(Ser380Arg), and p.(Val424Met). Sample names are shown on the X-axis. Scatter plot with a box and bar plot was drawn using the ScatterPlot.Bar website (https://scatterplot.bar/index.html, accessed on 23 August 2024). Groups that were not statistically significant were connected with lines and indicated as “ns”.

Luciferase activities of the p.(Val370Ala) and p.(Ser380Arg) variants were similar to the wildtype EDAR. These results were consistent with previous research findings [35,36], reaffirming that these mutations would likely not cause tooth agenesis. The p.(Met107Val) and p.(Ile130Asn) mutations showed reduced activities, while the p.(Val424Met) mutation exhibited a very weak signal (Figure 5).

### 3.5. Protein Structure Analysis

**Figure 6:**
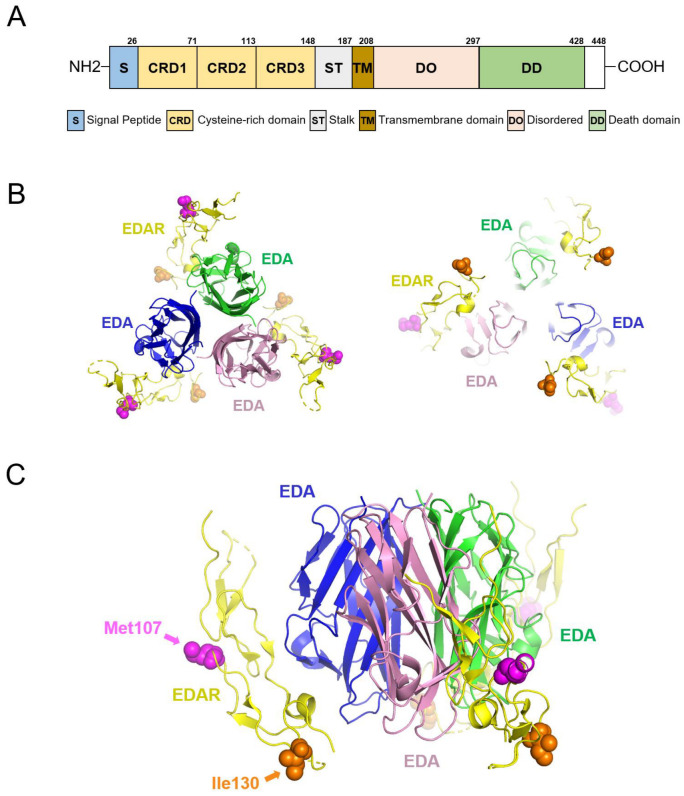
Gene diagram and 3D modeling. (**A**) Domain structure of EDAR. Amino acid numbers are shown above the diagram. Three cysteine-rich domains (CRD) bind with the EDA trimer. The death domain (DD) interacts with the EDARADD adapter protein to send signals to the downstream molecules. (**B**) The 3D modeling image of the EDA homotrimer interacting with EDAR CRD fragments. The left is the top view, and the right is the bottom view. Each EDA chain is indicated with the same color character, and EDAR fragments are shown in yellow. (**C**) An enlarged lateral view. Amino acid locations are indicated by arrows of the same color as the amino acid spheres (Met107 and Ile130).

The p.(Met107Val) and p.(Ile130Asn) mutations are located in the cysteine-rich domains (CRD), which interact with the exterior of the EDA homotrimer (Figure 6). However, these amino acid positions are not in direct contact with the EDA homotrimer. Therefore, the conformational changes in the CRD domains appear to reduce the binding efficiency to the EDA homotrimer [37].

The 3D protein analysis of other variants could not be performed due to the lack of crystal structure data. The p.(Val370Ala), p.(Ser380Arg), and p.(Val424Met) variants are all located in the death domain, which interacts with the EDAR-associated death domain (EDARADD).

## 4. Discussion

In this study, we analyzed three NSO families and identified recessive *EDAR* mutations. The proband of family 1 had two homozygous *EDAR* variants (NM_022336.4:c.319A>G p.(Met107Val) and c.1109T>C p.(Val370Ala)), but the p.(Val370Ala) variant was excluded based on previous research data and our luciferase activity results [24]. The proband also carried a heterozygous *WNT10A* variant (NM_025216.3:c.637G>A p.(Gly213Ser)), which is pathogenic in autosomal recessive oligodontia caused by *WNT10A* mutations [30]. The severe clinical phenotype of the proband could be explained by synergistic overlapping effects of having two mutations together, similar to recently reported cases of familial tooth agenesis with *LRP6* and *PAX9* mutations [31,32].

The father of the family 2 proband had no missing teeth but two misshapen teeth (peg lateralis of both maxillary lateral incisors). The paternal mutation was a novel missense mutation near the end of the death domain (c.1270G>A p.(Val424Met)), likely affecting interaction with EDARADD. This mutation showed almost no luciferase activity, suggesting a severe effect that may influence the formation of the father’s peg lateralis.

The mother of the family 3 proband was missing the right mandibular second premolar, and her maxillary lateral incisors were peg lateralis bilaterally. She had a heterozygous missense *EDAR* mutation (c.389T>A p.(Ile130Asn)) and a heterozygous *KREMEN2* mutation (NM_172229.3: c.1270C>T p.Arg424*). The *KREMEN2* mutation would produce a truncated protein lacking the last 39 C-terminal amino acids. The concomitant existence of these two mutations in the mother appears sufficient to cause single-tooth agenesis and peg lateralis formation, but not strong enough to cause oligodontia.

Due to study limitations, we could not fully elucidate the effect of the paternal intronic cis variants across exon 5 of the *EDAR* gene (c.[357-4G>A;c.440+50C>T]). However, our splicing assay revealed a cryptic 5′ splicing site in intron 5. This alternative splicing event would introduce an additional 100 bp intronic sequence after exon 5, resulting in a premature stop codon. Transcripts containing this premature stop codon would likely be degraded by the nonsense-mediated decay (NMD) surveillance system [38]. While these intronic variants do not directly produce truncated proteins, they appear to alter the splicing pattern and mRNA stability of the *EDAR* gene [39], potentially affecting its function in developmental processes [40]. Further studies employing techniques such as quantitative RT-PCR or RNA-seq would be beneficial to more precisely quantify changes in *EDAR* mRNA levels and splicing patterns in affected tissues.

## 5. Conclusions

In conclusion, we identified recessive mutations in the *EDAR* gene through comprehensive genetic analysis of three families with NSO. We elucidated the disease-causing mechanism through splicing analysis, luciferase assays, and protein 3D structure prediction. By synthesizing our findings with previous studies, we have demonstrated that caution is warranted when selecting candidate disease-causing genes, particularly when analyzing a single patient. This underscores the importance of thorough genetic screening and functional validation in multiple affected individuals or families.

## Figures and Tables

**Table 1:** Selective tooth agenesis categories.

Name	Abbreviation	Phenotype MIM Number	Inheritance	Gene	Gene/Locus MIM Number
Tooth Agenesis, Selective, 1, with or without orofacial cleft	STHAG1	# 106600	AD	MSX1	142983
Tooth Agenesis, Selective, 2	STHAG2	% 602639	AR		
Tooth Agenesis, Selective, 3	STHAG3	# 604625	AD	PAX9	167416
Tooth Agenesis, Selective, 4	STHAG4	# 150400	AD, AR	WNT10A	606268
Tooth Agenesis, Selective, 5	STHAG5	% 610926	AD		
Tooth Agenesis, Selective, 6, FORMERLY	STHAG6	# 601216	AR	LTBP3	602090
Tooth Agenesis, Selective, 7	STHAG7	# 616724	AD	LRP6	603507
Tooth Agenesis, Selective, 8	STHAG8	# 617073	AD	WNT10B	601906
Tooth Agenesis, Selective, 9	STHAG9	# 617275	AD	GREM2	608832
Tooth Agenesis, Selective, 10	STHAG10	# 620173	AR	TSPEAR	612920
Tooth Agenesis, Selective, X-linked 1	STHAGX1	# 313500	XLD	EDA	300451

**Table 2:** Mutagenesis primers.

	Forward Primer	Reverse Primer
c.319A>G p.(Met107Val)	5′-CACCAGGGGACGTGGAGAATGAC-3′	5′-GTCATTCTCCACGTCCCCTGGTG-3′
c.389T>A p.(Ile130Asn)	5′-CCGAGGAACAACTATGGCATG-3′	5′-CATGCCATAGTTGTTCCTCGG-3′
c.1270G>A p.(Val424Met)	5′-GGCTGGATGCTATGGAGTCCTTG-3′	5′-CAAGGACTCCATAGCATCCAGCC-3′
c.1109T>C p.(Val370Ala)	5′-CTGAGAAGGCTGCTGTGAAAACGTGG-3′	5′-CCACGTTTTCACAGCAGCCTTCTCAG-3′
c.1138A>C p.(Ser380Arg)	5′-CCTCGCCGAGCGCTTCGGCCTG-3′	5′-CAGGCCGAAGCGCTCGGCGAGG-3′

**Table 3:** In silico prediction and minor allele frequency of the variants.

	PolyPhen-2	Mutation Taster	CADD 1.7	dbSNP	Minor Allele Frequency
c.319A>G p.(Met107Val)	benign (score: 0.000)	polymorphism	14.25	rs61761321	0.003656
c.357-4G>A			1.835	rs748225	0.06239
c.442+50C>T			0.3	rs3749107	0.08188
c.389T>A p.(Ile130Asn)	probably damaging (score: 0.976)	disease causing (prob: 0.999)	24.4	rs764524798	0.00000
c.1270G>A p.(Val424Met)	probably damaging (score: 1.000)	disease causing (prob: 0.999)	28.6		
c.1109T>C p.(Val370Ala)	possibly damaging (score: 0.656)	polymorphism	27.6	rs3827760	0.039895
c.1138A>C p.(Ser380Arg)	probably damaging (score: 1.000)	disease causing (prob: 0.999)	28	rs146567337	0.001148

## Data Availability

The data presented in this study are openly available in ClinVar (http://www.ncbi.nlm.nih.gov/clinvar, accessed on 27 July 2024), Accession ID: SCV005088567.
